# Extensive Dural Venous Thrombosis With Associated Intracerebral Hemorrhage and Subarachnoid Hemorrhage Due to Protein S Deficiency: A Case Report

**DOI:** 10.7759/cureus.66803

**Published:** 2024-08-13

**Authors:** Abhinav K Rao, Thomas J Lee, Kody Dhaliwal, Nick Shaheen

**Affiliations:** 1 Department of Internal Medicine, Trident Medical Center, North Charleston, USA; 2 Internal Medicine, Rutgers University New Jersey Medical School, Newark, USA; 3 Department of Emergency Medicine, Trident Medical Center, North Charleston, USA; 4 Radiolgy/Interventional Radiology, Aultman Hospital/NEOMED, Canton, USA

**Keywords:** cerebral venous sinus thrombosis (cvst), thrombosis, acute subarachnoid hemorrhage, intracerebral hemorrhage, protein s deficiency

## Abstract

Cerebral venous thrombosis (CVT) is an uncommon but potentially severe condition, typically affecting younger individuals, pregnant women, and those with underlying thrombophilia. We report a rare case of a 63-year-old female who presented with altered mental status, facial droop, and slurred speech and was found to have an extensive dural venous thrombosis complicated by intracerebral and subarachnoid hemorrhage due to protein S deficiency. Given her diagnosis of protein S deficiency and thrombosis, careful anticoagulation was initiated, resulting in both clinical and radiographic improvement.

## Introduction

Cerebral venous thrombosis (CVT) is a rare entity, with an annual incidence of 1.32 per 100,000 person-years [1]. Furthermore, extensive CVT involving all cerebral veins and dural sinuses is rarely described in the literature, suggesting that its incidence is even lower. When CVT does occur, it is more commonly seen in patients aged less than 40 years, patients with thrombophilia, and pregnant women or women using hormonal contraceptives [2]. CVT is three times more likely to occur in women using oral contraceptives (OCPs) or pregnant women, indicating a strong association with estrogen [1]. Moreover, there are only a few reported cases of CVT due to protein S deficiency, as this disorder in itself is exceedingly rare [3].

## Case presentation

The patient was a 63-year-old female with a past medical history of hypertension, hyperlipidemia, and depression who presented to an outside hospital with altered mental status. She woke up one morning with a left-sided facial droop and slurred speech. She had gone to bed the previous night at 19:00 in her normal self. Over the past three days, there had been a gradual onset of slowed speech. Notably, she was not on any antiplatelet or antithrombotic medications and had no prior history of strokes. Additionally, her family history was significant for thrombosis, though the location was unspecified.

Upon arrival, the patient initially had unremarkable vitals, including normal blood pressure. Laboratory results were within normal limits. However, a CT scan of the head revealed a 2.2 x 1.9 x 2.7 cm right parietal lobe intracerebral hemorrhage with surrounding vasogenic edema, along with subarachnoid hemorrhage at the right parietal sulci and a subdural hematoma along the posterior falx, resulting in a 4 mm midline shift. Due to the severity of the hemorrhage, the patient was intubated and transferred to our medical center. On arrival, her systolic blood pressure was in the 150s. When her sedation was weaned off, her neurological exam showed spontaneous movement of both legs and the left upper extremity.

**Figure 1:**
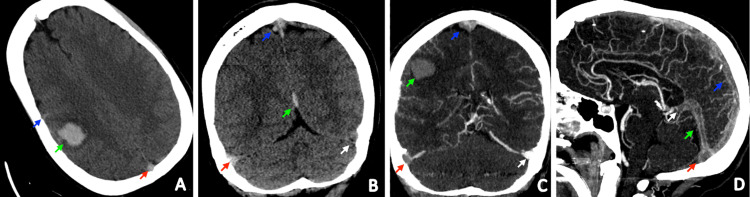
Extensive cerebral venous thrombosis with intracerebral and subarachnoid hemorrhage A. NECT axial image demonstrates a right perirolandic hematoma measuring 2.2 x 1.9 x 2.7 cm (green arrow). There is surrounding vasogenic edema. Adjacent sulcal hyperdensity likely reflects subarachnoid hemorrhage (blue arrow). Hyperdensity is present in the superior sagittal sinus (red arrow). B. Coronal NECT shows hyperdensity in the right transverse sinus (red arrow), superior sagittal sinus (blue arrow), and straight sinus (green arrow). Note that the density of the right transverse sinus is asymmetrically increased relative to the left (white arrow). C. Coronal CTV images demonstrate the right perirolandic parenchymal hematoma (green arrow) with adjacent vasogenic edema. Filling defects are present in the superior sagittal sinus (blue arrow) and right transverse sinus (red arrow) compatible with dural sinus thrombosis. Note that there is normal opacification of the left transverse sinus (white arrow). This explains the asymmetric hyperdensity on the NECT, which was secondary to the thrombus. D. Sagittal CTV images demonstrate filling defects involving the superior sagittal sinus (blue arrow), straight sinus (green arrow), confluence of sinuses (red arrow), and vein of Galen (white arrow), compatible with dural sinus thrombosis CTV: computed tomographic venography; NECT: non-contrast-enhanced computed tomography

Further imaging via CT angiography (CTA) revealed extensive intracranial venous thrombosis affecting multiple sinuses and veins, accompanied by a parenchymal right parietal lobe hemorrhage from the venous thrombus. Magnetic resonance angiography (MRA) of the neck was negative. Magnetic resonance venography (MRV) demonstrated diffuse thrombosis involving the superior sagittal sinus, straight sinus, right transverse sinus, right sigmoid sinus, and the vein of Galen with an associated subarachnoid hemorrhage and periolandic hematoma (Figure 1). At this point, given her lack of risk factors for induced hypercoagulability, an inherited disorder of hypercoagulability was suspected. The differential included protein C deficiency, factor V Leiden variant, protein S deficiency, and antithrombin deficiency, among other disorders of thrombophilia.

The patient was admitted to the ICU for the management of an extensive dural and sagittal CVT. The treatment included a heparin drip to maintain a target PTT of 50-70, a nicardipine drip to regulate blood pressure to maintain blood pressure between 120-140/60-80, and leviraticim 1g BID. Additionally, sodium levels were maintained above 145 with NaCl 3% boluses and Q.4h Na checks, while neuro checks were conducted hourly.

A multidisciplinary team including Neurosurgery, Cardiology, and ICU specialists collaborated on the patient's care. Incidentally, a large pericardial effusion and hiatal hernia were discovered on chest CT upon admission, requiring emergent pericardiocentesis. The patient underwent serial CT scans and was successfully extubated after six days. Improvement was noted in sinus flow after a week of heparin therapy, prompting the transition to warfarin. She completed treatment for suspected aspiration pneumonia and underwent a pericardial window due to re-accumulation of fluid. After discharge to a skilled nursing facility, she continued on warfarin and received follow-up care with hematology for hypercoagulability evaluation.

The patient remained on warfarin 2.5 mg daily after discharge. Her outpatient hypercoagulability workup six weeks after hospitalization revealed the following findings: protein S Ag, total - 50% (reference range: 60-150%); protein S Ag, free - 45% (reference range: 61-136%); protein S activity - 14% (reference range: 63-140%). She was diagnosed with protein S deficiency and continued on oral anticoagulation indefinitely. A follow-up non-contrast CT head scan one month later showed resolution of the intracranial hemorrhage and involutional encephalomalacia of the right parietal-occipital infarct. An MRV performed two and a half months post-discharge revealed an old area of infarction in the right parietal lobe, demonstrating appropriate evolution. Notably, the major venous structures of the brain remained patent.

## Discussion

Protein S deficiency is an autosomal dominant disorder characterized by a mutation in the PROS1 gene [4]. It can also occur due to acute illness, vitamin K deficiency, liver disease, chronic kidney disease, and chemotherapy [3-5]. Protein S is made in hepatocytes and endothelial cells and breaks down factors V and VII, which are implicated in clot formation. Dysfunctional and decreased levels of protein S result in uninhibited factors V and VII, leading to unchecked thrombosis in unusual locations, such as the cerebral veins [3,5].

CVT, as in this case, results in increased pressures in the capillaries and venules. Increased small vessel pressures result in ischemic injury with cytotoxic edema, and disruption of the blood-brain barrier with associated vasogenic edema. If the pressures are high enough, parenchymal hemorrhage can ensue due to venous and capillary rupture. Additionally, cerebrospinal fluid (CSF) absorption is impaired due to cerebral sinus obstruction. Impaired CSF absorption results in increased intracranial pressure (ICP), which can then lead to a vicious cycle that worsens pressures in the venules and capillaries, resulting in parenchymal hemorrhage and edema [2].

The clinical symptoms of CVT depend on the location of thrombosis. Classically, four syndromes have been described: isolated intracranial hypertension, focal neurological abnormalities, seizures, and encephalopathy. A patient may have one or more of these syndromes, as in this patient with thrombosis involving every cerebral vein, depending on the location and extent of thrombosis. Intracranial hypertension most commonly presents as a headache, with less common findings including visual deficits and papilledema [6,7]. Focal neurologic deficits most commonly involve hemiparesis and can be seen in 40% of patients with CVT. Seizures, experienced by 30-40% of patients, serve as an important diagnostic indicator, as they are less frequently observed in other types of strokes [6]. Patients who experience encephalopathy from CVT are more likely to be older [8].

Hypercoagulability thrombophilia laboratory workup should be initiated in all patients with CVT. D-dimer has low utility in the setting of CVT given the high false positive (9%) and false negative (24%) rates [7]. Importantly, hypercoagulability workup should be delayed until the acute illness is resolved, as this can obscure any interpretation of results. This patient was found to have protein S deficiency on an outpatient hypercoagulability workup, which is most commonly diagnosed with decreased free protein S levels on assays [3,4]. With regards to imaging, computed tomographic venography has high sensitivity and specificity in detecting CVT, and is comparable to MRI. While MRI with MRV exhibits slightly higher sensitivity, it requires more time and carries an increased risk of inducing nephrogenic fibrosis in patients with renal insufficiency due to the necessity for gadolinium contrast.

The purpose of anticoagulation in the management of CVT is to reduce thrombus extension, promote recanalization of occluded tracts, and prevent further thrombus formation. Unfractionated heparin titrated to twice the upper limit of normal has been shown in clinical trials to be superior to standard heparin titration [9]. Therapeutic low-molecular-weight heparin as a bridge to long-term vitamin K antagonists is another option. Furthermore, anticoagulation is indicated even in cases with associated hemorrhagic infarction as lysis of thrombus decreases venous outflow obstruction and decreases the risk of further hemorrhage. Additionally, no new hemorrhages or extension of hemorrhages were seen in patients started on anticoagulation in clinical trials [9,10]. Hence, careful and deliberate anticoagulation was applied in this case, resulting in thrombus recanalization and improvement in clot burden.

After initial heparinization, current guidelines recommend long-term anticoagulation with an oral vitamin K antagonist with an INR goal between 2.0 and 3.0 for three to six months in patients with provoked CVT. In patients with unprovoked CVT, oral anticoagulation should be continued for 6-12 months. Some patients may require lifelong oral anticoagulation, including those with recurrent CVT, DVT, PE, or thrombophilia, as in this case. The prognosis for CVT is good, with a majority (90%) of patients achieving complete or partial recovery [10].

## Conclusions

Protein S deficiency, a rare genetic disorder, can cause extensive CVT and related complications such as intracerebral and subarachnoid hemorrhage. Patients with unexplained thrombosis or CVT should undergo a thorough hypercoagulability workup to identify underlying conditions like protein S deficiency, guiding treatment decisions. Careful anticoagulation therapy in patients with CVT, even with associated hemorrhagic infarction, can promote vessel recanalization, limit thrombus extension, and improve clinical outcomes.
